# In-situ visualization of solute-driven phase coexistence within individual nanorods

**DOI:** 10.1038/s41467-018-04021-1

**Published:** 2018-05-02

**Authors:** Fariah Hayee, Tarun C. Narayan, Neel Nadkarni, Andrea Baldi, Ai Leen Koh, Martin Z. Bazant, Robert Sinclair, Jennifer A. Dionne

**Affiliations:** 1 Department of Electrical Engineering, Stanford University, 496 Lomita Mall, Stanford, CA 94305 USA; 2 Department of Materials Science and Engineering, Stanford University, 496 Lomita Mall, Stanford, CA 94305 USA; 3 Department of Chemical Engineering, Massachusetts Institute of Technology, Cambridge, MA 02139 USA; 4 DIFFER - Dutch Institute for Fundamental Energy Research, De Zaale 20, 5612 AJ Eindhoven, The Netherlands; 5 Stanford Nano Shared Facilities, Stanford University, Stanford, CA 94305 USA; 6 Department of Mathematics, Massachusetts Institute of Technology, Cambridge, MA 02139 USA; 7 Stanford Institute for Materials and Energy Sciences, SLAC National Accelerator Laboratory, Menlo Park, CA 94025 USA

## Abstract

Nanorods are promising components of energy and information storage devices that rely on solute-driven phase transformations, due to their large surface-to-volume ratio and ability to accommodate strain. Here we investigate the hydrogen-induced phase transition in individual penta-twinned palladium nanorods of varying aspect ratios with ~3 nm spatial resolution to understand the correlation between nanorod structure and thermodynamics. We find that the hydrogenated phase preferentially nucleates at the rod tips, progressing along the length of the nanorods with increasing hydrogen pressure. While nucleation pressure is nearly constant for all lengths, the number of phase boundaries is length-dependent, with stable phase coexistence always occurring for rods longer than 55 nm. Moreover, such coexistence occurs within individual crystallites of the nanorods and is accompanied by defect formation, as supported by in situ electron microscopy and elastic energy calculations. These results highlight the effect of particle shape and dimension on thermodynamics, informing nanorod design for improved device cyclability.

## Abstract (web summary)

Compared to thin films and other geometries, nanorods can exhibit particularly high performance in solute-intercalation-based energy and information storage devices. Here, the authors use in situ electron microscopy and spectroscopy to study the hydrogenation of palladium nanorods, revealing relationships between nanorod structure and device cyclability and capacity.

## Introduction

One-dimensional (1D) nanostructures such as nanowires and nanorods are becoming increasingly important components of batteries^1,2^, gas sensors^3,4^, solar cells^5–7^, transistors^8^, and light emitting diodes^9^. In energy-storing and information-storing, sensing and catalysis, nanorods enable better faradaic efficiency, improved reversibility, faster kinetics, and generally enhanced performance^2,4,10–12^. The superiority of nanowires is commonly attributed to their large surface-area-to-volume ratio, direct 1D electronic or ionic transport, and reduced likelihood of sintering compared to other nanoparticles^13–16^. For the particular case of solute-intercalation-based devices^17,18^, nanorods can be less prone to defect formation, likely due to their ability to relieve strain via lateral relaxation^19^.

A number of theoretical studies have explored solute-induced intercalation thermodynamics in nanorods and related 1D nanostructures^20–22^. Compared to nanoparticles (0D)^22–25^, thin-films (2D)^26^, and bulk (3D) systems, nanorods have been predicted to exhibit unique properties due to finite size effects. For example, in the 1970’s, it was predicted that first-order phase-change mechanism in a long-thin wire is energetically identical for both incoherent (i.e., defect-forming) and coherent pathways^20^. More recent theoretical studies have postulated that nanorods will preferentially form coherent phase-interfaces provided the diameter is below a critical value, regardless of length^19,21^. Related theory has suggested that defect formation is unfavorable in single-crystalline nanorods with sufficiently small diameters. Interestingly, the predicted critical diameter (~60–100 nm for 1% misfit)^19,21^ is over an order of magnitude larger than the critical thickness for thin films^27^. Still, reaction mechanisms in 1D nanostructures remain largely unknown, due to the difficulty in directly visualizing such transformations.

Here, we investigate solute-induced phase transformations in penta-twinned nanorods at the single-particle and sub-particle level, unraveling the influence of aspect ratio and twin defects on the thermodynamics. We take the hydrogenation of palladium as a model system. It is characterized by a first-order phase-transition from hydrogen-poor α-phase at low H_2_ pressures to a hydrogen-rich lattice-expanded β-phase at high H_2_ pressures. Using an environmental transmission electron microscope (TEM), we introduce hydrogen into the system and collect electron energy loss (EEL) spectra, selected area electron diffraction (SAED), and displaced aperture dark-field (DADF) images of single nanorods of lengths 45–550 nm and radii 7–16 nm in situ. By combining the local (1–3 nm) chemical composition from EEL spectroscopy and crystallographic information from SAED and DADF images, we find that the β-phase preferentially nucleates at the nanorod tips. With increasing hydrogen pressure, the β-phase front progresses along the rod length. Smaller nanorods with lengths less than 55 nm do not show coexistence of α and β phases, similar to single-crystalline Pd nanoparticles with sizes below ~200 nm^23,24^. However, longer nanorods do support stable phase coexistence and even multiple phase boundaries. This stable phase separation is present even at the single crystallite level, making the phase boundary continuous along the rod cross-section. Both elastic strain modeling and experimental dark-field microscopy suggest that such stable coexistence is supported by incoherent interfaces in these polycrystalline particles.

## Results

### Spatially localized single-nanorod isotherms

**Fig. 1:**
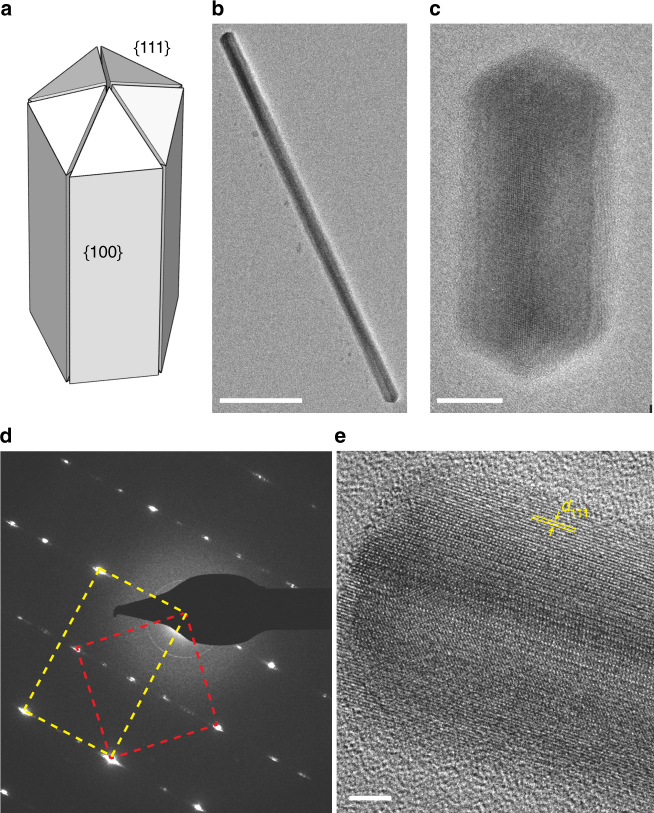
Pd nanorod schematic, micrograph, and diffraction pattern. **a** A three-dimensional view of a penta-twinned nanorod with (100) planes in the long edges and (111) planes at the tips. **b**,** c** TEM images of a long nanorod synthesized using seedless synthesis^28^ and a short nanorod synthesized using a seeded growth^29^. Scale bars are 100 and 10 nm, respectively. **d** Diffraction pattern of a representative penta-twinned nanorod, showing $\left\langle001 \right\rangle$ (red-dotted line) and $\left\langle112 \right\rangle$ (yellow-dotted line) zone axes. **e** High-resolution image showing (111) lattice fringes along the length of the nanorod. *d*_111_ = 2.2 Å. Scale bar is 2 nm

We begin by preparing penta-twinned palladium nanorods of varying length and aspect ratio. Nanorods were colloidally synthesized by reducing H_2_PdCl_4_ in the presence of CTAB as a stabilizer, resulting in penta-twinned rods (see Methods)^28,29^. Smaller nanorods of lengths 45–65 nm were synthesized using a seeded growth method, whereas longer nanorods of length 85–500 nm were synthesized by modifying a seedless growth method^28,29^. All radii were between 7–16 nm. Nanorods were dropcast onto a 20 nm SiO_2_ substrate and cleaned in the presence of oxygen-argon (1:3) plasma to remove organic ligands^22,23^. The SiO_2_ substrate minimizes contamination within the hydrogen environment and provides a featureless background for low-loss EEL spectroscopy^30^. Figure 1a includes a schematic and 1b, c show TEM images of two representative nanorods from the two different synthetic methods. The nanorod surfaces are (100) planes along the length (faces) and (111) planes at the tips. A SAED pattern in Fig. 1d shows the superposition of $\left\langle 001 \right\rangle$ (red square) from the bottom crystallite and $\left\langle112 \right\rangle$ (yellow rectangle) zone axis pattern from the top two crystallites^28,31,32^. The twinning of the crystallites is evidenced by the streaking of the diffraction pattern. The slight angular mismatch of the crystallites and hence slight splitting of the points is beyond the detector resolution, resulting in broadened diffraction point widths. The aberration corrected high-resolution TEM image in Fig. 1e shows (111) fringes (*d*_111_ = 2.2 Å) along the long axis of the rod, resulting from the $\left\langle112 \right\rangle$ zone axis.

**Fig. 2:**
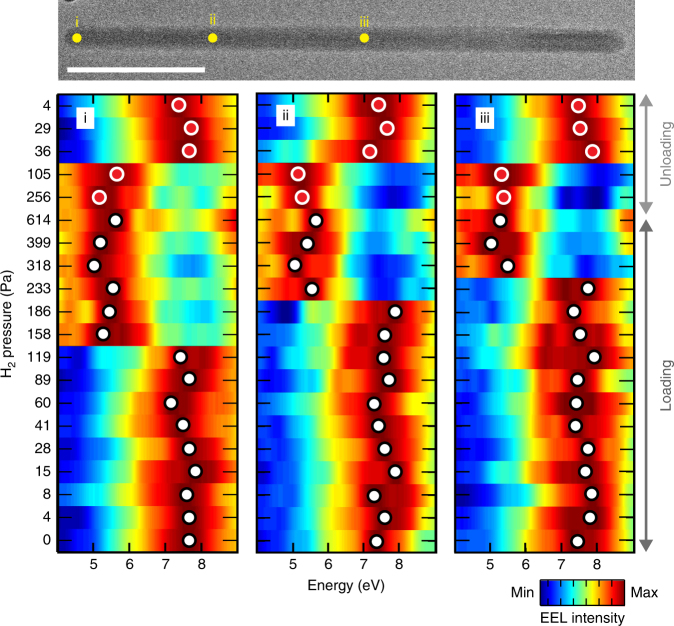
EEL spectra at different H_2_ pressures. Complete EEL spectra evolution over a full loading–unloading cycle for three points along the length of a 243 nm nanorod. The points where the spectra are collected are approximately marked in the TEM image of the nanorod at top. The corresponding H_2_ pressures are indicated next to each row. The spectra are smoothed with a locally weighted second-order polynomial (LOESS) regression and normalized by the height of the bulk plasmon resonance peak. The peak maxima are indicated with a white (loading) or red (unloading) circle. A shift of the bulk plasmon resonance indicates a phase-change. Scale bar is 100 nm

We study the single particle loading and unloading thermodynamics in situ using EEL spectroscopy to deduce the local phase. Notably, upon hydrogen absorption, the bulk-plasmon-resonance shifts from ~7.7 eV in the α-phase to ~5.6 eV in the β-phase^23^. We obtain absorption–desorption pressure-energy-loss isotherms at −27 °C by collecting low-loss EEL spectra at multiple points along the length of the nanorods with a focused beam (spot size ~1 nm), first with increasing and then with decreasing H_2_ pressure. Figure 2 presents the smoothed and normalized EEL spectra with varying H_2_ pressure at three different positions of a 243 nm long and 8.5 nm radius nanorod. Below 120 Pa, all positions’ spectra show peaks near ~7.7 eV, corresponding to the α-phase. At hydrogen pressures ranging from 158 to 233 Pa, we observe coexistence of the α-phase and β-phase in the particle. Positions along the rod-length hydrogenate at higher hydrogen pressures than the corner (position i), at 233 and 318 Pa, respectively, for positions ii and iii. Importantly, such two phase coexistence is observed independent of the time between acquisitions. While nanoparticle loading occurs within minutes^23,33^, we have waited from 15 min up to 105 min to ensure the system has reached a stable state (Supplementary Note 1).

### Mapping length-dependent phase coexistence

As Fig. 2 illustrates, the nanorod tip appears to hydrogenate at a lower pressure than the rest of the particle. We studied 34 nanorods of lengths 45–550 nm using EEL spectroscopy, of which 29 showed stable phase coexistence.

**Fig. 3:**
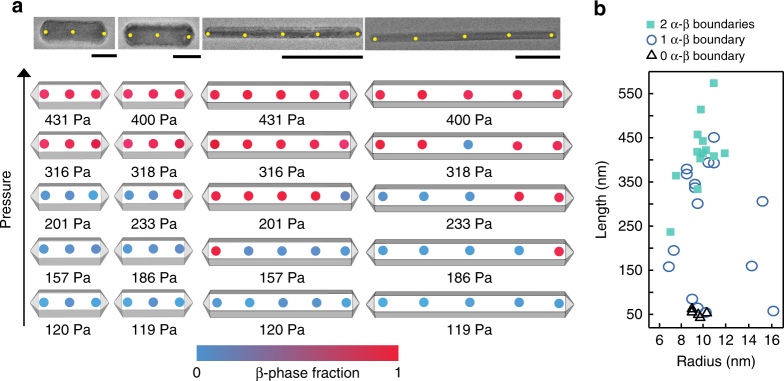
Gradual loading of Pd nanorods. **a** Loading states of four nanorods are shown at different hydrogen pressures. The color of the points shows the β-spectral contributions of the point spectra (calculated using MCR) collected at that position of the nanorod. The β-phase is indicated by red and α is indicated by blue. The scale bar is 20 nm for the first two columns and 100 nm for the last two. **b** 34 nanorods showing that nanorods shorter than 55 nm (with ~10 nm radii) tend not to support phase coexistence, whereas nanorods longer than 250 nm are more likely to support two phase boundaries

Short nanorods are less likely to support phase coexistence than longer nanorods. We probe seven nanorods of length less than ~60 nm and radii ~10 nm, of which five show no phase coexistence. TEM images and local phase (β spectral weights calculated using multivariate curve resolution (MCR): see Methods and ref. ^34^) of two representative nanorods of lengths 51 and 55 nm are shown in Fig. 3a. The first nanorod hydrogenates fully in one pressure step similar to the behavior of single-crystalline particles with edge lengths below ~200 nm^23,24^. In contrast, the second nanorod hydrogenates at the tip at 233 Pa and fully hydrogenates at 318 Pa.

Phase coexistence is more common for longer nanorods, with the number of phase boundaries dependent on length. We investigated 26 nanorods with lengths ranging from 85–550 nm and radii 7–16 nm, all of which show stable phase coexistence. We observe two types of behavior: in the third column of Fig. 3a, the β-phase nucleates at one of the tips below 157 Pa; the phase-transition progresses toward the other tip until full hydrogenation at 316 Pa. On the other hand, in the last nanorod of Fig. 3a, the β-phase nucleates first at one tip at 186 Pa and then at the other tip at 318 Pa. For all 29 nanorods showing coexistence, hydrogenation starts at the tip region and creates one or two phase boundaries depending on length. We find that, longer nanorods (*L* ≥ 250 nm) tend to support two phase boundaries at higher pressures whereas shorter nanorods (*L* ≤ 250 nm) support either zero or one phase boundary as shown in Fig. 3b. The higher probability of forming two β-phase nuclei in the long nanorods indicates either that the physical dimensions of these longer rods exceed the long-range H–H elastic interaction in Pd, or that the defects inherent in the rod decouple the two nuclei (Supplementary Fig. 6 shows one such defective rod).

**Fig. 4:**
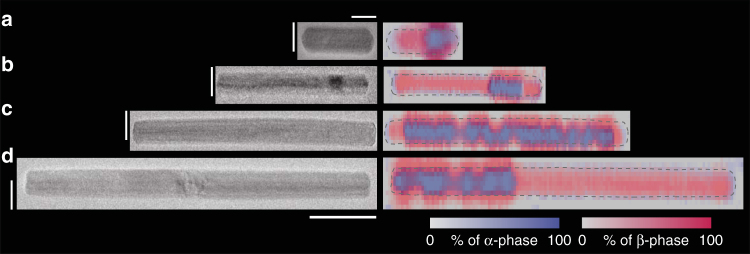
Phase progression along the length of nanorods. **a**–**d** TEM images and EEL spectral images of four different nanorods at 100 K while cooling started from 254 Pa. The collected EEL spectral images have been decomposed into constituent spectra using MCR and the α-spectral and β-spectral weights are superimposed to produce the figures. The nanorod boundaries are drawn approximately as a guide to the eye. The scale bars correspond to 20 nm for (**a**) and 50 nm for (**b**–**d**), respectively in *x*-axis. Along *y*-axis, scale bar is 20 nm

To image the phase distribution with better resolution, we obtain “frozen” EEL spectral images with sub-5 nm spatial resolution. Here, we cool the system to 100 K while slowly removing the hydrogen, thus effectively trapping the hydrogen inside the nanoparticles (see Methods). Figure 4 shows four representative particles with overlaid α and β spectral contributions calculated using MCR from the EEL spectral images. In Fig. 4a, we capture a short nanorod of length ~60 nm after nucleation at one tip and at almost 50% new phase fraction. For longer nanorods, we capture the rods in different stages of loading. Figure 4c shows a state just after nucleation at both corners and Fig. 4b, d show cases of two and one phase boundaries, respectively. As suggested by our point measurements (Fig. 3) and confirmed by frozen EEL spectral images, the β-phase establishes planar phase boundaries after nucleation to minimize total free energy through minimizing the interfacial surface area.

### Length-independent tip nucleation

Our isotherms and EEL spectral maps indicate that nanorod tips generally transform at lower pressures than the rod interior or rod edges. Indeed, all 29 rods showing phase coexistence exhibit tip loading. The preferential nucleation at the tips can be due to several factors, including their high curvature and the tip surface crystallography. In particular, the tip curvature locally reduces the coordination number of Pd atoms, which can make H-adsorption more likely^35^. In addition, the nanorod tips are (111)-terminated, in contrast to the (100) rod edges. H-atoms can directly diffuse into an octahedral site for Pd(111) once adsorbed, whereas for Pd(100), H-atoms first occupy a tetrahedral position before hopping to an octahedral site. Finally, from surface-wetting theory of coherent nucleation, the nucleation barrier decreases with wetted surface area to strained volume ratio, which is minimized at the nanorod tips and remains fairly constant for all particles^36^. In fact, for nanorods of lengths 85–500 nm, we observe tip nucleation at almost the same pressure regardless of rod length (Supplementary Fig. 5), likely due to the constant energy penalty paid (*kT* ln *P*) to support one α*-*β boundary. In addition, the thermodynamic (full) loading pressure has positive correlations with radius and surface-area and a weak dependence on length, as seen in Supplementary Fig. 5; this result can be attributed to the interplay between surface stress from the hydrogenated surface layer, inherent strain of the penta-twinned particles and the longitudinal elastic energy contribution from the flat-phase boundary^23,24,37^.

### Phase coexistence and reduced miscibility within individual crystallites

**Fig. 5:**
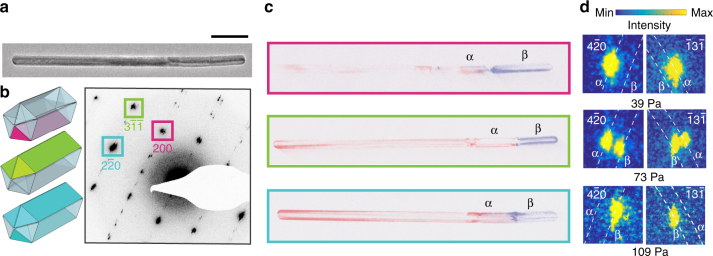
Diffraction and DADF images revealing phase separation within single crystallites. **a**,** b** TEM image of a representative nanorod and corresponding selected area diffraction pattern at 120 Pa. Scale bar is 100 nm. The diffraction pattern shows the splitting of points indicating coexistence of phases with two different lattice parameters. **c** False-colored DADF images collected from 200, $3\bar 1 \bar 1$, and $2\bar 20$ points. The corresponding crystallites are colored in **b**. For each point marked in **b**, the dark-field images are collected by selecting the inside points for β-phase (blue) and the outside points for α-phase contributions (red). **d** Zoomed in $4\bar20$ and $\bar13\bar 1$ diffraction points of another nanorod in a dehydrogenated state at 39 Pa, in a coexisting state at 73 Pa and in a hydrogenated state at 109 Pa

We find that the observed phase coexistence occurs even within the rod’s constituent crystallites using DADF imaging at −27 °C. The lattice expanded β-phase has a larger lattice constant than the α-phase^20,33^; accordingly, during coexistence of α-β-phases, each point radially splits into two in the diffraction plane (intensity distributions in two different scattering rings corresponding to α and β lattice parameters) as seen in Fig. 5b for the nanorod in Fig. 5a. We use the objective aperture to select one of the paired spots and construct dark-field images depicting each phase contribution. In Fig. 5c, taken at 120 Pa, the dark-field image from the 200 point shows α-β phase coexistence in the bottom crystallite, whereas the image from the $3\bar 1\bar 1$ point shows phase coexistence in the top two crystallites. This behavior is in contrast with the phase coexistence shown in three dimensionally confined polycrystalline icosahedra, where the twin-boundaries act as elastic-strain relaxation centers with each individual crystallite exhibiting a pure phase^38^. Both of the dark-field images as well as the image from the $2\bar 20$ point (which is in both zone axes, $\left\langle001 \right\rangle$ and $\left\langle {112} \right\rangle$) show that all three crystallites have a near-identical α and β phase distributions; accordingly, the phase-front progression with pressure is nearly the same for all three crystallites.

We verify that all five crystallites change phase together by collecting diffraction patterns with varying hydrogen pressures at −27 °C. Figure 5d shows representative diffraction points from the top two crystallites (the $\bar13\bar1$ point) and from the bottom crystallite (the $4\bar20$ point) at hydrogen pressures of 39, 73, and 109 Pa. The white lines indicate the calculated position for the α-phase and β-phase diffraction spots. At 39 Pa, both the $4\bar20$ and $\bar 13\bar 1$ peaks are centered on the α-phase radius, whereas at 73 Pa, both of the points show splitting along the radial direction, indicating coexistence of the two phases in the top two and the bottom crystallites. At 109 Pa, the transformation completes and both points are centered at the β-phase position. These data support our conclusion that the diffracting three crystallites and (very likely) all five crystallites change phase together.

Finally, we note that upon hydrogenation, the lattice parameter from individual crystallites within our nanorods increases by 2.8–3.5% with respect to the lattice parameter at 0 Pa (Supplementary Fig. 4). Compared to a bulk expansion of 3.5%, this smaller expansion points toward reduced hydrogen miscibility in nanorods^38,39^. This result can be attributed to the compressive strain at the nanorod core, owing to their 7.5° angular deficit^40,41^. Similar compressive strain contributes to the non-hydrogenated cores in icosahedral nanoparticles as well^33^.

### Critical radius and length

Coherency strain at the phase boundary can be relaxed by creating defects. To determine whether the phase coexistence is coherent or accompanied by defects, we model the elastic strain energy and the energy penalty for a dislocation creation in a cylindrical rod (Supplementary Note 2). In particular, we calculate the critical radius for dislocation formation by modifying calculations of single-crystal heterojunction nanowires^19,21^ to account for the reduction of distance of a dislocation from a free surface due to the twin boundaries. Using this model, we find that, for rods with radii greater than 10 nm, the elastic strain energy reduction upon forming a defect exceeds the energy required to form a defect. Accordingly, rods with radii above 10 nm will tend to exhibit multiple phases accompanied by defect formation. Our experimental DADF images acquired during coexistence show crystallographic dislocation lines at multiple positions of the nanorod including the phase boundary (Supplementary Fig. 3). Such dark-field images, together with the calculated critical radius, suggest defect-mediated phase coexistence, and hence a semi-coherent interface.

In addition, our model shows that, for a fixed radius, beyond a critical length, nanorods are unlikely to support a coherent interface. For nanorods above the critical length, even if there is an initial coherent interface, it will energetically be more favorable to support a defect after a certain volume fraction of the new phase (Supplementary Eqs. 10 and 12). We approximate this critical length to be lower than 50 nm for 10 nm radius. In nanorods longer than twice this critical length, it can be energetically favorable to form two defects after a certain volume fraction, explaining our observation of longer nanorods supporting two phase boundaries. The presence of edge dislocations near phase boundaries has also been reported in LiNi_0.5_Mn_1.5_O_4_ (LNMO) nanoparticles^42^, whose thermodynamics is similar to Pd. Ulvestad et al. also reported phase coexistence in large Pd nanocrystals when defects are formed, similar to our rod study^24^. Our results generally imply a defect supported phase coexistence, which can result in reduced cyclability of the system.

## Discussion

In an ideal Pd bulk system, the H-induced coherency strain results in a macroscopic energy barrier, thus preventing phase coexistence in equilibrium^43^. The presence of twin-boundaries, creation of defects and nano-confinement effects serve to reduce this coherent energy barrier^23,24^. However, it is unlikely that the elastic energy will be monotonically increasing or decreasing, completely eliminating the barrier. Thus, we postulate that the coexisting states are due to both thermodynamic contributions (reduction of the energy barrier) and different kinetic pathways resulting into trapped energy-minima. The time scales of hydrogenation in the nanorods are at least 2–3 times slower than cubes studied at identical conditions, which indicate an increase of diffusion activation energy by at least 1.6 times. Accordingly, previous papers have correlated slow diffusion with compressive strain (as is present in nanorod cores), twin-boundaries, and dislocations^44^.

In conclusion, we have directly visualized the hydrogen-induced phase transition of penta-twinned palladium nanorods spanning a broad size range. Rods shorter than 55 nm only occasionally show phase coexistence while longer rods exhibit one or two phase boundaries depending on length. Nucleation of the hydrogenated phase always begins at the rod tips. Interestingly, the nucleation pressure is constant for all rods irrespective of aspect ratio, implying a nearly constant energy penalty for a phase boundary. Diffraction and dark-field imaging reveal that the rod’s constituent crystallites change phase in a concerted fashion and suggest that the phase boundary is continuous throughout the nanorod cross-section, despite the twin-boundaries. Further, dark-field imaging and free-energy minimization calculations imply defect formation at the phase boundary. Together, our results suggest that elastic strain relaxation dominates the nucleation and growth process in nanorods. Accordingly, the most promising penta-twinned nanorod geometries for devices are those with aspect ratios less than ~ 3 and radii below 10 nm; such small-radii nanorods should result in a coherent, defect-free phase transition. These results are complementary to, but distinct from the thermodynamics observed for 0D, 2D, and 3D systems: while coherent transitions are seen for dimensions below 200 nm in nanoparticles, they are seen for thicknesses well below 10 nm^45^ in thin films. In addition, the twin-planes do not serve as phase boundaries as they do for multiply-twinned icosahedra, most likely due to the longer length of the crystallites. The observed phase separation can be extended to other metal-hydrides and Li-ion battery electrode systems with similar thermodynamics. Developing and optimizing methods to synthesize small-radii rods will enable improved cyclability and device lifetimes in a variety of applications, including energy and information storage, and chemical sensing.

## Methods

### Synthesis of 40–60-nm long Pd nanorods

40–60 nm penta-twinned nanorods are synthesized by a seeded growth method following previously published procedures^29^. Typically, a seed solution of Pd small nanoparticles was prepared by adding 12.5 mL of a 1 mM Na_2_PdCl_4_·3H_2_O solution to 25 mL of 0.15 M CTAB solution at 30 °C. Then, 3 mL of an ice-cold 0.01 M NaBH_4_ solution was quickly added under vigorous stirring. The palladium suspension was stirred for 15 min and then removed from heat. The seed solution was used 2 h after its preparation.

For the growth solution, a 25 mL of 3 mM Na_2_PdCl_4_·3H_2_O solution was mixed with a 25 mL of 0.24 M CTAB solution under gentle stirring at 30 °C in a 50 mL centrifuge tube. Two such solutions were made. After 5 min of mixing, the solution turns turbid. One milliliter of a 0.08 M sodium ascorbate solution was then added to each of the reaction mixtures. Finally, 90 and 270 μL of the seed solutions were injected into this growth solution. The reaction was allowed to proceed for 3 h.

To clean the CTAB capped nanoparticles, 1 mL of each reaction mixture was centrifuged at 5000 rpm for 10 min. After removing the supernatant, the particles were resuspended in 1 mL of 1 μM CTAB solution. This process was repeated two times. After third cleaning cycle, the particles were redispersed in 450 μL of 1 μM CTAB solution.

### Synthesis of 100–500-nm long Pd nanorods

Pd penta-twinned nanorods of length 100–500 nm were synthesized by modifying previous reported method^28^. In a typical synthesis, 450 μL of 10 mM H_2_PdCl_4_ was added to 15 mL of 100 mM CTAB at 95 °C under gentle magnetic stirring (400 rpm). The H_2_PdCl_4_ solution was prepared by suspending 8.9 mg PdCl_2_ (50.2 μmol) in 208 μL of 0.48 M HCl (100 μmol), sonicating for ~10 min and then diluting the solution to 5 mL with water. After gently mixing the solution, 300 μL of 100 mM freshly prepared KI solution and 360 μL of 10 mM AgNO_3_ were added in sequence. Five minutes later, 150 μL of 100 mM freshly prepared L-Ascorbic acid was added to the solution. The reaction was kept in 95 °C oil bath for 1 h and the stirring was stopped after 3 min of adding ascorbic acid. To clean the nanoparticles, the same method as the previous synthesis was followed (cleaned 3 times), however the centrifuge speed was 4000 rpm and each centrifugation was done for 10 min. The synthesis results nanorods with a wide length distribution; lengths ranging in 100–200 nm, but of fixed radii (10 ± 2 nm).

For longer nanorods of lengths 300–400 nm, we changed the temperature to 90 °C in the above described synthesis and the reaction was kept at 90 °C for 80 min. Reducing the temperature reduces the reduction rate and results in longer nanorods. The resultant nanorods have similar radii ranging from 9 to 13 nm.

The purification of these longer particles was as follows: four 1 mL aliquots of the reaction mixture were centrifuged at 4000 rpm for 10 min. After removing the supernatant, 250 μL of 1 μM CTAB was added to each tube and the aliquots were combined, and centrifuged at 4000 rpm for 10 min. The supernatant was removed, 1 mL of 1 μM CTAB was added, and the particles were centrifuged at 4000 rpm for 10 min. The supernatant was removed and 450 μL of 1 μM CTAB was added.

### TEM grid preparation

Seventy-five microliters of the two long Pd nanorod solutions and 20 μL of the two growth solutions (shorter Pd nanorods) were combined in a centrifuge tube. A 20-nm thick silicon dioxide grid (TEMWindows, SO100-A20Q33) is plasma cleaned for 1 min in an air plasma at 10 W and 10 μL of the mixture is dropcasted onto the grid. After 7 min, the remaining liquid is wicked off with a kimwipe.

### Experimental conditions

The general experimental conditions are very similar to the previously published papers^23,33,38^. The TEM grid with Pd particles is cleaned for 1 min in an 3:1 Ar/O_2_ plasma with RF power of 50 W. The grid is then mounted into a TEM cryo holder (Gatan, Inc.), which has a temperature controller that can allow control of temperature with a precision of ±0.1 K. The Titan TEM (FEI) has ETEM capabilities which allows us to vary the H_2_ pressure from 4 to ~600 Pa, using a home-built gas cart with mass-flow controllers. In order to avoid condensation of contaminants on the sample during hydrogen gas flow, we use a liquid nitrogen cooled cold finger during our environmental TEM experiments, which minimizes beam-induced hydrocarbon contamination during STEM-EELS and SAED data acquisition at all hydrogen pressures. We obtain our EEL spectra and diffraction while operating the microscope at 80 kV, which gives higher interaction cross-section, and high-resolution TEM images at 300 KV. The H_2_ (99.9999%, Praxair) pressure in the microscope chamber is monitored using an Edwards Barocell 600 capacitance manometer with a precision of 4%.

### EEL spectroscopy isotherms

From our previous experiments on Pd nanocubes^33^, we have an estimate of the operating temperature range to ensure full hydrogenation below the pressure limit of the column, according to the vant Hoff relation: ln *p* ∝ −1/*T*^23^. We operate at a temperature of 246 K (−27 °C) to ensure that complete loading/unloading isotherms can be obtained. EEL spectra were collected with a focused beam at 80 kV operating voltage in mono-chromated STEM mode (Monochromator spot size 15) using a 50 μm C3 aperture, 48 mm camera length, a 2.5 mm spectrometer entrance aperture, and at a dispersion of 0.01 eV per channel. This setting corresponds to a convergence semi-angle of 8.7 mrad and a collection semi-angle of 20.2 mrad. The exposure time is limited to 0.04 s. The beam is parked away from the particle while not collecting spectra to minimize beam induced heating. EEL spectra are shifted by 3.5 eV to exclude the zero-loss peak (ZLP) from the field-of-view to avoid CCD saturation during the acquisition time.

At the beginning of the experiment, we heat up the sample to 60 °C under hydrogen pressure of 400 Pa for ~20 min to minimize any oxide layer that may have formed while loading the sample into the TEM holder and microscope. We then slowly cool down the sample to our operating temperature of −27 °C under 400 Pa of hydrogen and verify that the particles are in their β-phase by collecting EEL spectra. We then pump hydrogen gas out of the microscope to let the nanorods fully desorb. These two steps ensure the particles are cycled at least once to relax possible stresses built in the nanoparticles during synthesis^23^. A set of particles is the marked in the sample and are tracked along the whole experiment. After these initial steps, we start increasing the hydrogen pressure in the microscope column up to 600 Pa in multiple steps. At each pressure point, after the pressure has stabilized inside the column, we wait for at least 30 min before taking EEL spectra. Any time when the spectrum is not recorded, the beam is parked away from the particle.

### EEL spectral maps in frozen state

EEL spectral maps with resolution ~5 nm helps us to unravel the α-β phase distribution during transition. The EEL spectral maps are collected by freezing the sample to avoid electron beam induced local heating and possible desorption during the long acquisition time required. We first increase the hydrogen pressure to ~230 Pa, and then wait ~10 min for loading to occur. Next, we reduce the temperature to 180 K and slowly remove the hydrogen from the column. The system finally reaches to 100 K, which is well below the temperature at which the palladium surface becomes catalytically inactive (220 K)^46^. Therefore, we effectively trap hydrogen inside the palladium matrix, enabling high-resolution mapping. We then obtain EEL spectral images by focusing the STEM beam and taking an area containing the particle, which we divide into a grid with a spatial resolution of around 3–5 nm. Each point spectrum is taken with exposure time of 0.04 s and the spectral image takes 10–25 min depending on the nanorod length. To calibrate the spectra, we aligned the first and last spectra of each row with SiO_2_ bandgap of 10.6 eV and assume a linear drift in between.

### Diffraction based isotherm

All of the diffraction patterns were collected at 80 kV using a 180 nm selected area aperture. To minimize beam-induced heating, we limit the electron dose rate to 10 electrons per square angstrom per second. Each diffraction pattern is acquired for 1 s. We start by heating the sample to 60 °C in a hydrogen pressure of ~400 Pa to get rid of any oxide layer that may have formed at the surface due to air exposure during sample loading. We then slowly cool down the sample to our operating temperature of −27 °C under 400 Pa of hydrogen, and record the diffraction patterns of the particles in their β-phase. From our EEL spectroscopy isotherm measurement, we know that these temperature and pressure conditions ensure full loading of our nanoparticles. We then pump hydrogen gas out of the microscope to let the nanorods fully desorb and collect α-phase diffraction patterns at 0 Pa. These α-phase diffraction patterns provide the reference interplanar distances for our isotherm calculations, and help us confirm that the particles were indeed hydrogenated in the previous step (thus, cycled). After these preliminary steps, we acquire equilibrium diffraction isotherms by increasing the hydrogen pressure to 600 Pa in multiple steps. At each step, we wait for 30 min after the hydrogen pressure stabilizes inside the column to make sure the system has reached near equilibrium.

The positions of all available diffraction spots at 0–600 Pa are fitted using a two-dimensional voigt function. The 0 Pa diffraction pattern at −27 °C is used as the reference pattern to determine the change in distances between a pair of diffraction points (for example  $11\bar1$, 200 etc). The bottom crystallite of the nanorod diffracts along $\left\langle {001} \right\rangle$-zone axis. We measure the distance between an opposing pair of 200 points and use that to plot the diffraction isotherms for the bottom crystallite. Similarly, the top crystallite isotherm is calculated from the percentage change of the distance between $3\bar1\bar 1$ points.

### DADF imaging

Similar to the procedure used to acquire EELS isotherms, we cycle the nano-particles once before conducting our measurements. We then go to ~100 Pa slowly, as from our previous EELS experiments, we expect coexisting states near this pressure. Electron dose rate is 10 e/Å^2^s. We wait for 10 min before probing. We use the 180 nm selected area aperture and the acquisition time for each diffraction pattern is 1 s.

At ~91 Pa, some particles show splitting of each diffraction point into two indicating the coexistence of α and β phases. We bring the 10 μm objective aperture and collect dark-field images from each constituent spot. When we collected dark-field images from 200, $3\bar1\bar 1$, and $2\bar20$ points, we note that two particles desorbed hydrogen at the end due to this continuous beam exposure. The dark-field images were collected at an exposure of 2.55 s at the CCD.

To collect more dark-field images, we unloaded the particles and slowly increased the hydrogen partial pressure to 174 Pa. Then we waited for 10 min and cooled the system to 180 K, and slowly removed the hydrogen so that the system eventually cools down to 100 K (similar to EELS spectral imaging). We then collect dark-field images as described before.

### EEL spectra fitting

To fit the EEL specta and spectral images, we use MCR based on ref. ^34^ (open-source MATLAB package). We fit each spectrum as a linear combination of four basis spectra. The α and β basis spectra are collected at the middle of many particles at 4 Pa and at 600 Pa, respectively, and analyzed following  these steps: (i) we consider spectral range of 4–10 eV and then normalize, and average over multiple particles and collections, (ii) we collect background spectra away from the particles at hydrogen pressures of 4 and 400 Pa. These spectra are fitted using nonlinear fitting into a double exponential curve (ZLP and hydrogen peak at ~13.6 eV). They will be used as corresponding background spectra in the next step. (iii) We fit a weighted summation of a voigt spectrum and the corresponding background spectrum from step (ii) to the experimental spectra of step (i). These fitted voigt spectra will be used as basis spectra for α and β phases.

Using a similar method, we obtain a β-phase transverse surface plasmon basis spectrum by fitting a voigt function to an average of 10 experimental spectra collected by putting the beam aloof near the middle of the particle. The fourth basis spectra used for MCR fitting is the background spectra with hydrogen.

The error function of MCR is **E** = **D** − **CS**^**T**^, where **D** is a *m* × *n* matrix containing *m* spectra with *n* points; **C** is an *m* × *p* matrix containing the spectral weights of the *p* different components for each of the *m* spectra, **S** is an *n* × *p* matrix containing the spectra of the *p* pure components, and **E** is a residuals matrix^33,34^. By minimizing **E**, we obtain an optimized concentration profile **C**, which describes the whole dataset better. We do the fitting for energy range of 4–10 eV, to exclude the ZLP tail and high hydrogen absorption tail which peaks at 12.6 eV. For the spectral images, **E** is minimized for the whole 3D dataset together.

The spectral weights plotted in main text are calculated by dividing β-spectral weight at a point by the summation of all four basis spectral weights.

### Data availability

All relevant data are available from the corresponding authors upon request.

## Electronic supplementary material

Supplementary Information
