# Supplementary material for: In-situ visualization of solute-driven phase coexistence within individual nanorods
Source: Nat Commun. 2018 May 2;9:1775. doi: 10.1038/s41467-018-04021-1 (PMC5932065; doi:10.1038/s41467-018-04021-1)
Supplement: Supplementary file 1 — Supplementary Information [file 41467_2018_4021_MOESM1_ESM.pdf]

In-situ visualization of solute-driven phase coexistence within individual nanorods

Hayee et al.

1 Supplementary Note 1: Time dependence of coexisting states

To verify the stability of the coexisting states under e-beam irradiation, we collect EEL spectra at different times after increasing the H_2 pressure. During the experiments, the beam is parked away from the particle if we are not collecting EEL spectra. Supplementary Fig. 1(a) presents one case where the nanorod reaches equilibrium at 15 minutes and does not change state until 105 minutes at 112 Pa. From our previous studies on Pd nanocubes [1], we also found that the particles reached equilibrium before 30 minutes. Based on previous results and this time-dependent study, we chose 30 minutes as our waiting period after pressure stabilization at every pressure point of isotherm experiments.

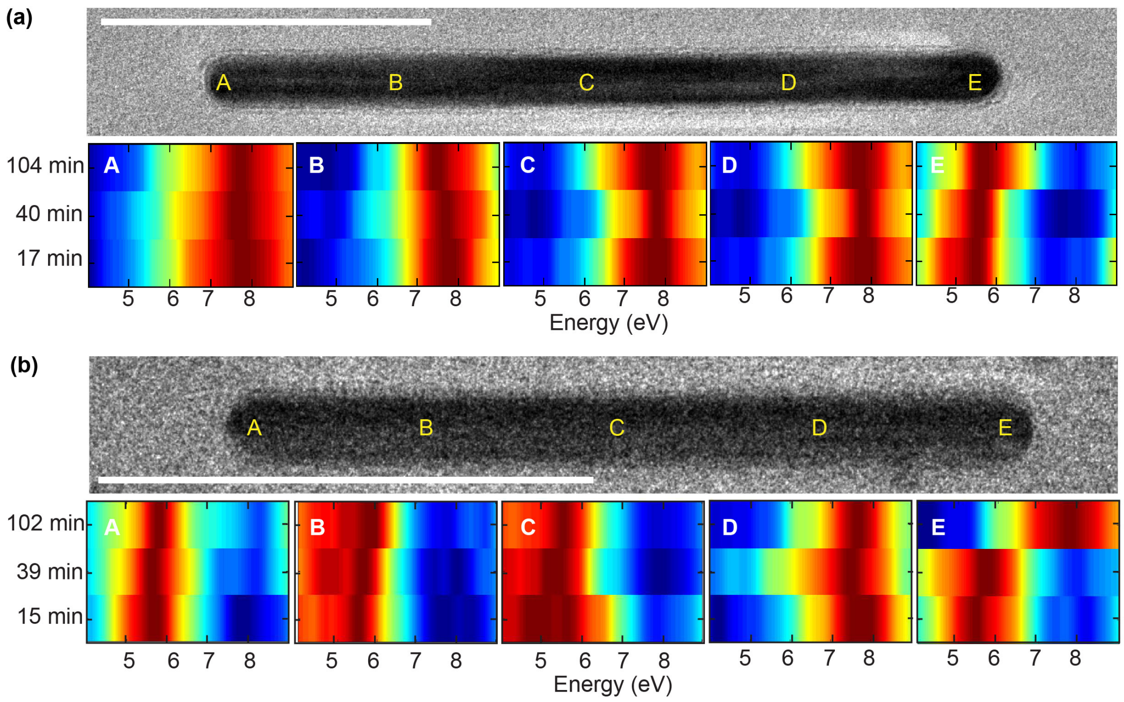

Supplementary Figure 1: **EEL spectra at different time intervals** TEM image and point EEL spectra collected at different points of two different nanorods at different times after stabilization of pressure. (a) shows a particle which retains its state after three measurements, however (b) shows beam induced unloading at the corner. However, case in (b) is rare and happened in 1 particle among 8 observed particles. Scale bars are 100 nm.

In our experiment, five nanorods retain coexistence after multiple beam exposures, however, one nanorods shows unloading at its corner point after third beam exposure at 105 minutes (shown in Supplementary Fig. 1(b)). Additionally, two more particles switched from coexisting state to completely hydrogenated state at $\sim$100 minutes in the third acquisition. These changes can be due to multiple acquisition in the particles. To minimize beam induced loading and unloading, we immediately remove the beam from the particle after EEL spectra collection and work at low magnifications, as well as avoid taking double spectra at any single point for EEL spectral isotherms. However to collect one EEL spectra, the beam was on the particle for around $1-3$ seconds as this is limited by an operator physically moving the probe away.

We confirm that these coexisting states are not due to our dose rate (i.e., multiple electron beam exposures), and that these particles do not fully hydrogenate at a given pressure with extended time periods. In a separate experiment, we set the pressure and then collect our first spectra after 100 minutes. We probe four particles at 112 Pa; three of the particles show coexistence. One nanorod is still unloaded, which is likely due to the pressure being low for that particular rod nucleation. This experiment demonstrates that the coexisting states are long-lived compared to nanocubes, triangles [2] and octahedra (under review), all of which completely hydrogenation within 30 minutes or less under the same experimental conditions.

2 Supplementary Note 2: Energy with incoherent phase boundary

Composition with one incoherent phase-boundary Lets assume that the particle is a cylinder with radius r_0 and length L. Palladium hydride is isotropic and the misfit is purely dilatational. Therefore, the Bitter-Crum theorem can be used to calculate the strain energy of the mixture of the two phases [3, 4]. Given a volume fraction v of the β phase, the strain energy of the system is given by,

$$W_e = 2G\frac{1+\nu}{1-\nu}(\varepsilon^0)^2(c_\beta - \bar{c})(\bar{c} - c_\alpha)V \tag{1}$$

$$= \frac{E}{1-\nu}(\varepsilon^0)^2(c_\alpha - c_\beta)^2 v(1-v)V, \tag{2}$$

where $\bar{c} = c_\beta v + c_\alpha(1-v)$ and G, E, and ν are the shear modulus, Young's modulus and Poisson's ratio respectively. Let the incoherent interface create a relaxation due to a plastic strain of $\varepsilon_p = 4b/(2\pi r_0)$ where b is the magnitude of the Burger's vector $\mathbf{b}$. Therefore, the new misfit strain is $\varepsilon' = \varepsilon^0 - \varepsilon_p$. The new misfit strain will give rise to a strain energy,

$$W_e' = \frac{E}{1-\nu}(\varepsilon')^2(c_\alpha - c_\beta)^2 v(1-v)V, \tag{3}$$

The plastic strain caused by edge dislocations along the interface will produce its own elastic

field whose energy is given by [5],

$$W_d = \gamma \varepsilon_p^2 A, \tag{4}$$

where,

$$\gamma = \frac{E r_0 (1 - \nu \cos^2\theta)}{8(1-\nu^2)} \left(1 + \ln\frac{\bar{h}}{b}\right), \tag{5}$$

with $\bar{h} = \min(L,\, 2r_0/\pi)$. Area A is the cross-sectional area and should not be confused with the surface area S. Now, the conversion from a coherent to an incoherent phase boundary will occur only when the energy of the latter is smaller than the former, or $\Delta W = W_e' + W_d - W_e < 0$. Therefore,

$$-\frac{E}{1-\nu}[(\varepsilon^0)^2 - (\varepsilon')^2](c_\alpha - c_\beta)^2 v(1-v)V + \gamma\varepsilon_p^2 A < 0. \tag{6}$$

On rearranging, substituting $V/A = L$ (for a cylinder) and noting that $v(1-v) \le 1/4$, we obtain

$$\frac{\gamma}{EL} < \frac{[(\varepsilon^0)^2 - (\varepsilon')^2](c_\alpha - c_\beta)^2 v(1-v)}{\varepsilon_p^2(1-\nu)} \le \frac{[(\varepsilon^0)^2 - (\varepsilon')^2](c_\alpha - c_\beta)^2}{4\varepsilon_p^2(1-\nu)} \tag{7}$$

$$= \frac{(\varepsilon^0 + \varepsilon')(c_\alpha - c_\beta)^2}{4\varepsilon_p(1-\nu)}. \tag{8}$$

When a coherent interface is formed, the interfacial energy is low and the strain energy is stored in the bulk through long-range interactions. However, the opposite happens in the case of an incoherent interface. The incoherent interface stores all the energy near the surface and a smaller strain energy permeates through the bulk. Here, $\frac{\gamma}{EL}$ is a non-dimensional number that compares the interfacial energy to the bulk energy. As can be seen, when L is small, the ratio is large which implies that the interfacial energy will be much larger than the bulk strain energy, and hence a coherent interface would be preferred. Whereas, when L is large, the opposite is true, which is confirmed from the experiments. The critical length when the interface changes is given by,

$$L_{\text{cr}} = \frac{4\varepsilon_p \gamma (1-\nu)}{E(\varepsilon^0 + \varepsilon')(c_\alpha - c_\beta)^2}. \tag{9}$$

Below L_{cr}, an incoherent interface will not be formed. For a given length above L_{cr}, the volume fraction at which phase boundary transition occurs can be computed by solving the quadratic equation,

$$v^2 - v + \frac{\varepsilon_p \gamma (1-\nu)}{EL(\varepsilon^0 + \varepsilon')(c_\alpha - c_\beta)^2} = 0 \quad \text{or} \quad v^2 - v + \frac{1}{4}\frac{L_{\text{cr}}}{L} = 0. \tag{10}$$

The transition will occur at the smaller of the two roots of the quadratic equation.

Composition with two incoherent phase-boundaries For the case of having two phase-boundaries, we can calculate the volume fraction v' needed to support a second phase boundary. Assuming isotropy, the following equation must be satisfied:

$$-\frac{E}{1-\nu}[(\varepsilon^0)^2 - (\varepsilon')^2](c_\alpha - c_\beta)^2 v'(1-v')V + 2\gamma\varepsilon_p^2 A = 0. \tag{11}$$

Inserting the value of v computed from (10), we obtain

$$v'(1-v') = \frac{1}{2}\frac{L_{\text{cr}}}{L} \quad \text{or} \quad v'^2 - v' + \frac{1}{2}\frac{L_{\text{cr}}}{L} = 0. \tag{12}$$

Now, as we have assumed that a single phase boundary already exists, we have $L > L_{\text{cr}}$. If $L < 2L_{\text{cr}}$, the second incoherent boundary will not be supported, and if there is no additional nucleation, the equilibrium mixture will have only one incoherent phase boundary. In the event that another phase nucleates, the particle will be fully in β-phase. However, if $L > 2L_{\text{cr}}$, the second phase boundary will form at a volume fraction given by (12).

Calculating critical length and radius In our previous section, we used an analytical expression for elastic strain very similar to that of two-dimensional films [6]. As the exact solution of elastic strain energy for a 1D case is not trivial, we approximate the strain energy for a misfitting layer of length L on top of a semi-infinite whisker with a radius of r_0 following Ref. [5]. The misfit strain between the whisker and the mismatched layer is ε_0. The total strain energy stored in the semi-infinite nanorod system with a layer of L' height on top ($-\infty < z < L'$) can be approximated as: [5]

$$W_e(\varepsilon_0) = \frac{E}{1-\nu}\frac{\pi r_0^2 L' \epsilon_0^2}{1+A_\nu \rho}, \tag{13}$$

where E is Young's moduli, ν is Poisson's ratio, $\rho = L'/2r_0$ and A_ν is a fitting parameter ($A_\nu = 27.3 \pm 0.55$ for $\nu = 1/3$). In this equation, a first-order approximation of the volume fraction contribution to the energy has been used, as compared to the semi-infinite rod length, the new misfitted phase length (L') is much smaller i. e. , $v \ll 1$.

The excess energy of the state with a dislocation pair with respect to a fully coherent state can be written by following [5]:

$$\Delta W(r_0, h) = \frac{E r_0}{1-\nu}\left[\frac{1}{1+A_\nu \rho}\pi r_0 L' \left(\frac{4b_{\text{eff}}^2}{\pi^2 r_0^2} - \frac{4b_{\text{eff}}}{\pi r_0}\epsilon_0\right) + C\left(1 + \ln\frac{\bar{h}}{b}\right)\right], \tag{14}$$

where $\bar{h} = \min(L',\, 2r_0/\pi)$, $C = (1-\nu\cos(\theta)^2)b^2/[2\pi(1+\nu)]$ and $\rho = 2L'/r_0$. Solving Eq. (14) for $L' = \infty$ gives the critical radius (r_{cr}) of the system, for which any length of mismatched layer can be supported on top. On the other hand, solving for $\Delta W = 0$ for a radius above critical radius gives the maximum length supported before creating defects (L'_{cr}).

We use (14) to calculate the critical radius for coexistence of Pd and PdH_x in a nanorod. For hydrogen concentration, $x = 0.6$ in PdH_x for β phase in bulk, the lattice mismatch ε_0 is $\sim 3.5\%$. The burger's vector for FCC crystal is commonly along $1/2 < 110 >$ for slip planes of $\{111\}$. BV can also be typically along $1/3 < 111 >$ for Frenkel's dislocations. The penta-twinned nanorods have (111) planes along the length, thus any lattice mismatch at the interface of the two phases should result in a dislocation loop along $< 111 >$. We take $\theta = 90^o$ and b_{eff} = b for pure edge dislocations and Poisson's ratio, $\nu = 0.39$. Solving Eq. (14) for $L = \infty$, we get critical radius of 13 and 16 nm for BV along $1/3 < 111 >$ and $1/2 < 110 >$ respectively.

However, one of the critical differences between a penta-twinned nanorod and a single-crystal nanorod is the existence of five twin boundaries, which can act as strain relaxation points. If we consider these twin boundaries as 'free-surfaces' similar to lateral surfaces, then the average distance between a dislocation pair from any free surface changes significantly. We define the minimum distance between an edge dislocation and a'free surface' as

$$f(r) = \begin{cases} r_0 - r & \text{if } \; r_0 - r < r\tan\theta \\ r\tan\theta & \text{if } \; r_0 - r > r\tan\theta, \end{cases} \tag{15}$$

where r_0 is the radius of the nanorod. To calculate the average minimum distance for a pair of dislocations, we average $f(r)$ over the cross-sectional area of a crystallite:

$$\bar{h} = 2 * \frac{5}{\pi r_0^2} \int_0^{\tan 36^\circ} \int_0^{r_0} f(r) r d\theta dr. \tag{16}$$

Numerically solving, we get $\bar{h}$ as $0.2661 r_0$. With this condition, the critical radius becomes 13 and 10.6 nm for $\mathbf{b} = 1/2 < 110 >$ and $\mathbf{b} = 1/3 < 111 >$, respectively. The values are very near to the radii of our observed nanorods ($8 - 12$ nm). The model does not take into account the radial strain distribution inherent to a penta-twinned structure due to the 7.5^o angle deficit when five tetrahedra come together [7], so most-likely over-estimates the critical radius.

We can also equate Eq. 14 to zero and calculate the maximum length of the new phase allowed for radii above r_{cr}. Supplementary Fig. 2(c) shows the critical length that can be supported before creating dislocations for different nanorod radii and different conditions in Eq. 14. Near the critical radius, the critical length has a rapid fall-off for a radius change < 1 nm, which can explain 2 nanorods in the length range of 48-65 nm and radius $\sim$10 nm support coexistence and 5 do not. From the main text Fig. 3b, we see that one nanorod with length 58 nm shows coexistence which is due to its radius (16.5 nm) being well above the critical radius. We also plot our experimental data on top of the plot with green circles depicting nanorods without phase-coexistence and orange squares for nanorods with α-β coexistence. From Supplementary Fig. 2(c), we see that for $A_v =$ 24, the critical length for a radius of 10 nm is $\sim$ 23 nm, where from our experiments, we expect it to be $\sim$ 55 nm. From our theoretical analysis, nanorods longer than $2L_{\text{cr}}$ can support two interfaces. However, from our experiments, we see nanorods longer than 250 nm tend to support two boundaries. Thus, our approximate model show similar trend of the data, however, the critical length from the model falls off more rapidly than the data, which is due to the fitting parameter A_v used in Ref. [5]. A later paper using a detailed FEM model for single-crystal nanorods proves that this 'fall-off' of critical length above critical radius is indeed much-flatter and shows similar trend as our experimental data [8]. However, a FEM modeling with twin-boundaries is beyond the scope of the paper.

Lastly, Schwarz et al. [3] discussed that there is an energy barrier for equilibrium coherent co-existence of phases in bulk. Accordingly, our calculations and dark field images suggest formation of defects to support the observed coexistence. With defect formation (incoherent transformation)

and nano-confinement, the energy barrier for coherent phase-coexistence decreases, as calculated by Ulvestad et al. [9]. However, the chemical potential curve should still be non-monotonic as a function of phase fraction, preventing phase-coexistence in a true equilibrium state. This implies that kinetic limitations must play a role in explaining the observations. The defects and dislocations may act as hydrogen traps, causing kinetic limitation to diffusion as discussed in Ref. [10, 11].

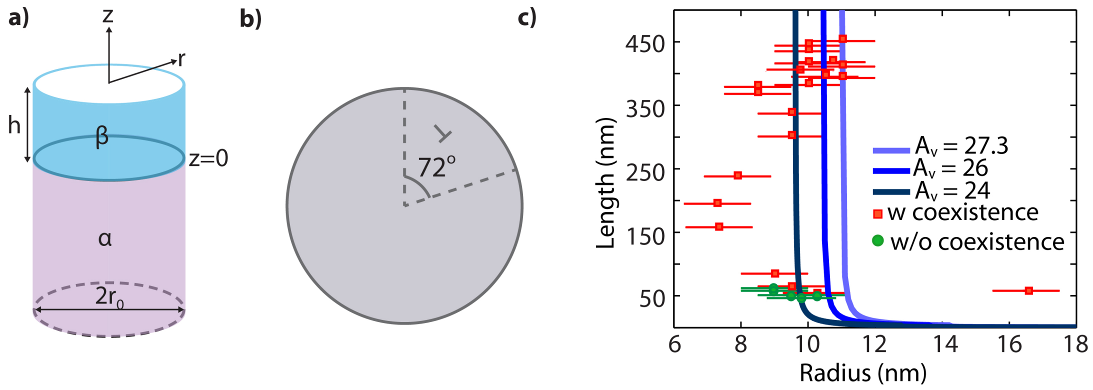

Supplementary Figure 2: **Critical thickness calculation.** (a) The lattice mismatched β-phase layer of height h is in equilibrium on top of a infinite rod in α-phase of radius r_0. (b) Cross-section of a nanorod showing the five twin boundaries. The average of the minimum distance of a pair of dislocation from a twin-boundary or lateral surface is calculated over the area of a single crystallite. (c) Critical length variation with nanorod radius assuming five -twin boundaries and pure edge dislocation formation for lattice misfit of 3.5% and ν = 0.39. The experimental observations with phase-coexistence are plotted in orange squares and without coexistence is plotted with green circles.

3 Supplementary Note 3: Dislocation loops in dark field images

The diffraction pattern shows discernible splitting when there is coexistence of α-β phases. We collected bright field (BF) and DADF images at coexisting state after freezing the system to 100K after being at 114 Pa for 10 minutes. The DADF images from the whole diffraction points (inside and outside spot corresponding to β and α phases) show parallel crystallographic lines with opposite contrast in BF and DF at the phase boundary, as well as some other positions along the rod. In Supplementary Fig. 3(a), the DADF image collected at $\vec{g} = 020$, shows a dark line at the phase boundary (the boundary is marked by the purple arrow), which has an angle of 44° with 110-direction. We know, the angle between 110 and 100-directions is $45°$. The crystallographic nature of the lines point towards the contrast originating from dislocations loops at the phase-boundary. Similarly Supplementary Fig. 3(b) show dark lines at the phase boundary for DADF images collected for $\vec{g} = 2\bar{2}0$. The line has an angle of 64° with nanorod growth direction, which can indicate the plane being $(3\bar{1}\bar{1})$.

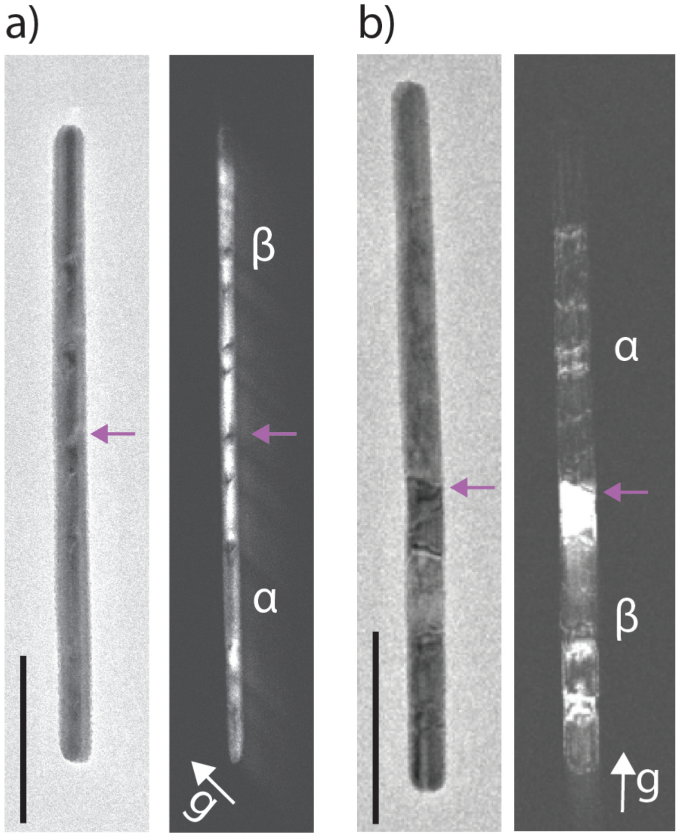

Supplementary Figure 3: **Phase boundary and defects in dark field images.** Bright field and DADF images showing complementary contrast at the phase boundary for images collected along (a) $\vec{g} = 020$ and (b) $\vec{g} = 2\bar{2}0$ for two different nanorods.The dark lines at the phase boundary are approximately 2-3 nm thick at 80 kV. Scale bars are 100 nm.

4 Supplementary Note 4: Diffraction-lattice-parameter isotherms

We obtain diffraction patterns of the nanorods at varying hydrogen pressures at a temperature of $-27°$C. The diffraction points are fitted with a two-dimensional voigt function and the distance between two pair of points is calculated. The distance between the corresponding planes is inverse of this distance. We calculate the change of lattice parameter with varying hydrogen pressures and use that to plot the pressure versus lattice parameter change isotherms. Supplementary Fig. 4(a) and (b) show such isotherms for two different nanorods. For the first nanorod, we plot the change of lattice parameter for $(11\bar{1})$ and (200) type points, originating from the top two and the bottom crystallites, respectively. The change in lattice parameter is calculated with respect to the lattice distance at 0 Pa. For the second nanorod, the isotherms are for $(2\bar{2}0)$ (from both zone axes) and for $(3\bar{1}\bar{1})$ planes, respectively. In both nanorods, we see the lattice expansion in β phase is less than the 3.5% observed in bulk. Lattice expansion is a good measure of hydrogen miscibility in the solid, thus, this indicates that hydrogen miscibility in penta-twinned nanorods is less than that of bulk. This can be due to the compressed core of the nanorods.

The second nanorod in Supplementary Fig. 4 show coexistence of α and β phases at an intermediate pressure of 180 Pa before full loading. At this stage, the diffraction pattern has split-diffraction points because of the two phases with different lattice parameter. Due to the difficulty of fitting such diffraction spots, we exclude that point from Supplementary Fig. 4. This complements the EEL spectroscopy data presented in main text Fig. 3, where the phase progression for the whole nanorod is plotted.

5 Supplementary Figures

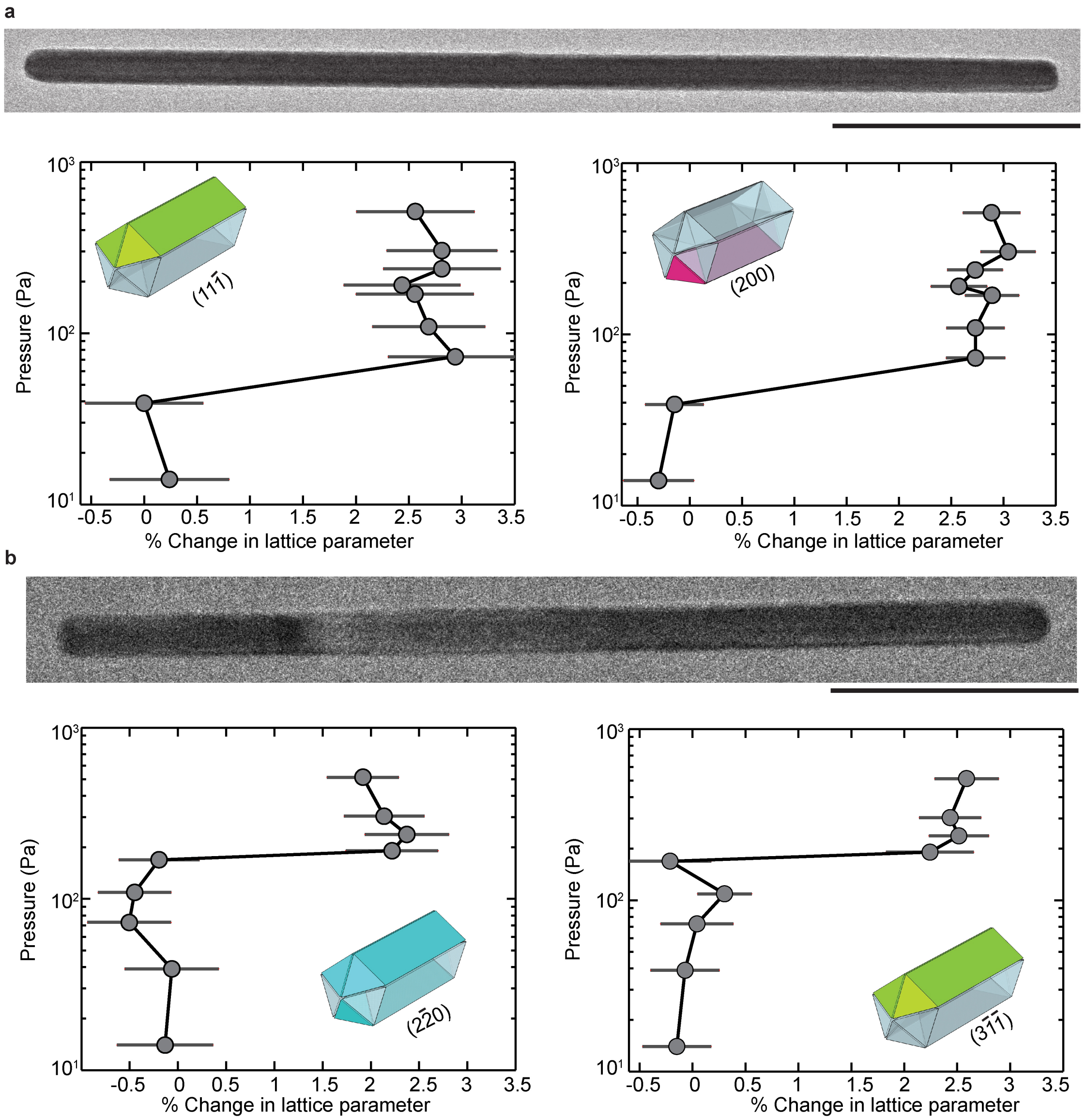

Supplementary Figure 4: **Pressure-lattice-parameter isotherms.** TEM image and and diffraction-lattice parameter expansion isotherms for $\langle 112 \rangle$ and $\langle 100 \rangle$ zone axes of two nanorods. The error bars are calculated using fitted voigt function widths and following standard error propagation rules. Scale bar is 100 nm.

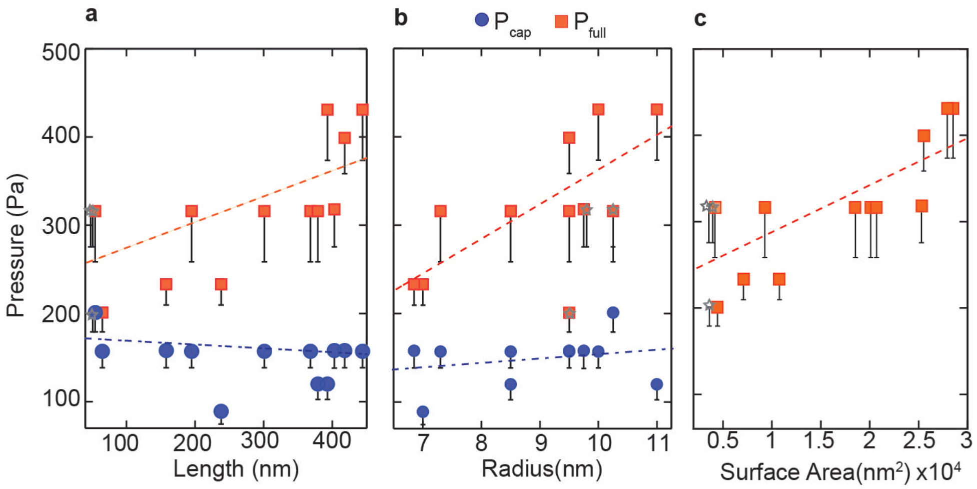

Supplementary Figure 5: **Loading pressure dependence on nanorod dimensions.** Tip and full loading pressure dependence on nanorod (a) length and (b) radius. (c) Full-loading pressure dependence on rod surface-area. The star marks are for the particles which hydrogenate in one step. These particles are excluded for the 'tip loading'. The error bars for y-axis are drawn from the mid value between two observed pressure points to the pressure at which the loading occurred. The dashed lines are guides for the eye.

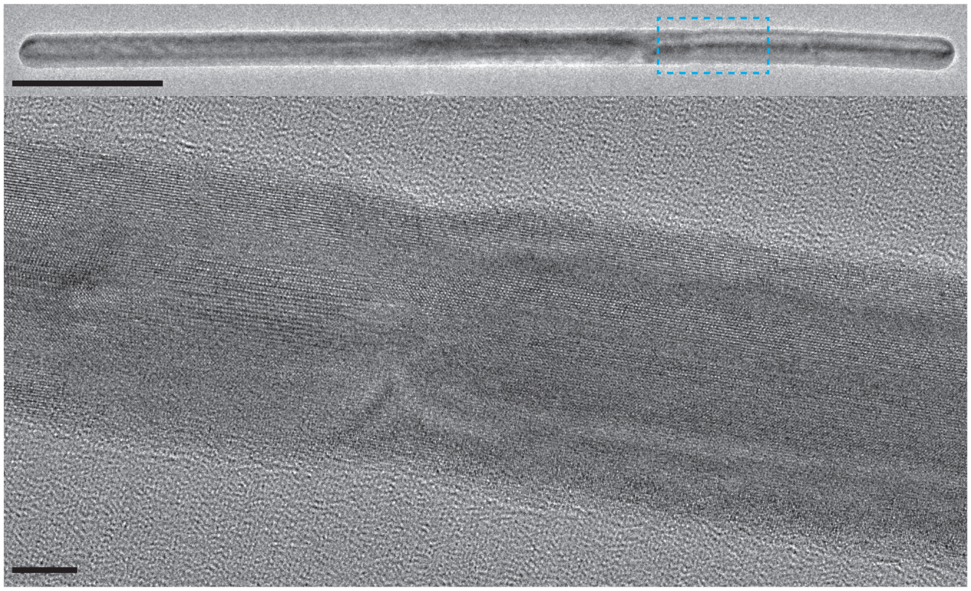

Supplementary Figure 6: **Inherent defects in long nanorods.** (a) TEM image of a 628 nm long nanorod. Scale bar is 100 nm. (b) HRTEM image of the boxed area (approximate) showing lateral defects. Scale bar is 5 nm. These defects can contribute towards the stable α-β phase coexistence.

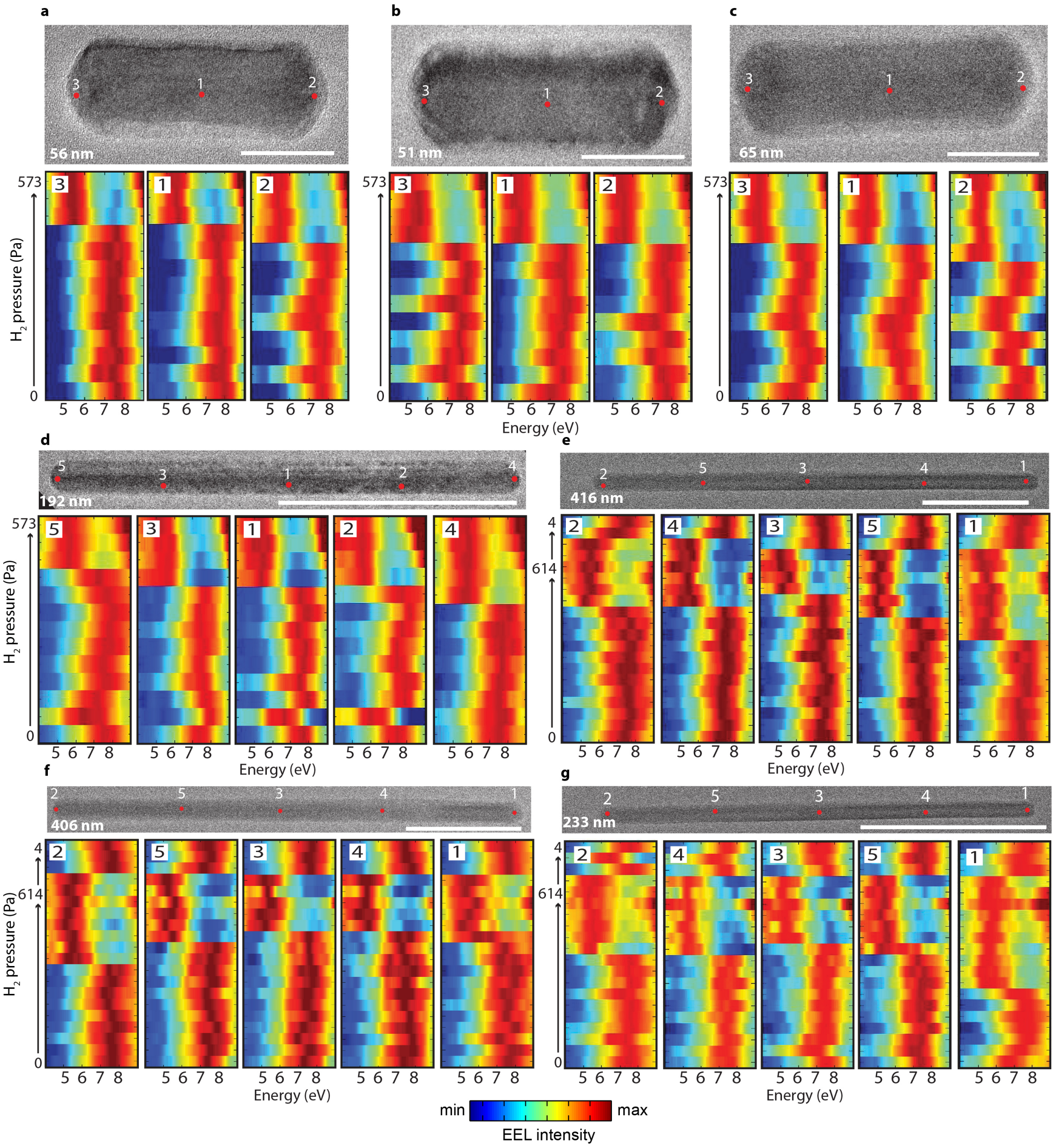

Supplementary Figure 7: **Pressure dependent EEL spectra of palladium nanorods** Colormap of the STEM-EEL spectra acquired at increasing H_2 pressures over different positions of seven representative nanorods. The respective TEM images are shown on top and approximate beam positions are marked by red circles. The EEL spectra are smoothed using a locally weighted 2nd order polynomial (LOESS) regression and normalized to the strongest resonance peak between the α and β phases. Particles in (a), (b), (d) and (e) are the ones in main text Fig. 3 panels (i), (ii), (iii) and (iv) respectively. The pressure values in the y-axis for particles (a-d) are 0, 4 ,8, 14, 27, 59, 85, 120, 156, 201, 316, 431, 573 Pa. The pressure values for panels and (d-g) are 4, 8, 15, 28, 41, 60, 89, 119, 158, 186, 233, 318, 399, 614, 256, 105, 36, 29, and 4 Pa. The numbers on the TEM images are the sequence at which the spectra are collected at each pressure.

References

1. Narayan, T. C., Baldi, A., Koh, A. L., Sinclair, R. & Dionne, J. A. Reconstructing solute-induced phase transformations within individual nanocrystals. *Nature Materials* (2016).

2. Baldi, A., Narayan, T. C., Koh, A. L. & Dionne, J. A. In situ detection of hydrogen-induced phase transitions in individual palladium nanocrystals. *Nat. Mat.* **13**, 1143–1148 (2014).

3. Schwarz, R. B. & Khachaturyan, A. G. Thermodynamics of open two-phase systems with coherent interfaces: Application to metalhydrogen systems. *Acta Materialia* **54**, 313–323 (2006).

4. Schwarz, R. & Khachaturyan, A. Thermodynamics of open two-phase systems with coherent interfaces. *Physical Review Letters* **74**, 2523 (1995).

5. Glas, F. Critical dimensions for the plastic relaxation of strained axial heterostructures in free-standing nanowires. *Physical Review B* **74**, 121302 (2006).

6. Matthews, J., Jackson, D. & Chambers, A. Effect of coherency strain and misfit dislocations on the mode of growth of thin films. *Thin Solid Films* **26**, 129–134 (1975).

7. Niekiel, F., Spiecker, E. & Bitzek, E. Influence of anisotropic elasticity on the mechanical properties of fivefold twinned nanowires. *Journal of the Mechanics and Physics of Solids* **84**, 358–379 (2015).

8. Ye, H. *et al.* Critical thickness and radius for axial heterostructure nanowires using finite-element method. *Nano Letters* **9**, 1921–1925 (2009).

9. Ulvestad, A. *et al.* Three-dimensional imaging of dislocation dynamics during the hydriding phase transformation. *Nat. Mater.* **16**, 565–571 (2017).

10. Oriani, R. A. The diffusion and trapping of hydrogen in steel. *Acta Metallurgica* **18**, 147–157 (1970).

11. Di Leo, C. V. & Anand, L. Hydrogen in metals: A coupled theory for species diffusion and large elastic–plastic deformations. *International Journal of Plasticity* **43**, 42–69 (2013).
